# The laboratory of the 1990s—Planning for total automation

**DOI:** 10.1155/S1463924692000105

**Published:** 1992

**Authors:** Linda A. Brunner

**Affiliations:** Development Department Pharmaceuticals Division CIBA-GEIGY Corporation Ardsley New York USA

## Abstract

The analytical laboratory of the 1990s must be able to meet and accommodate the rapid evolution of modern-day technology. One such area is laboratory automation. Total automation may be seen as the coupling of computerized sample tracking, electronic documentation and data reduction with automated sample handling, preparation and analysis, resulting in a complete analytical procedure with minimal human involvement. Requirements may vary from one laboratory or facility to another, so the automation has to be flexible enough to cover a wide range of applications, and yet fit into specific niches depending on individual needs.

Total automation must be planned for, well in advance, if the endeavour is to be a success. Space, laboratory layout, proper equipment, and the availability and access to necessary utilities must be taken into account. Adequate training and experience of the personnel working with the technology must also be ensured. In addition, responsibilities of installation, programming maintenance and operation have to be addressed. Proper time management and the efficient implementation and use of total automation are also crucial to successful operations.

This paper provides insights into laboratory organization and requirements, as well as discussing the management issues that must be faced when automating laboratory procedures.


Journal of Automatic Chemistry, Vol. 14, No. 2 (March–April 1992), pp. 43–45

### The laboratory of the 1990s—Planning for total automation

**Linda A. Brunner**

*Development Department, Pharmaceuticals Division, CIBA-GEIGY Corporation, Ardsley, New York, USA*

*The analytical laboratory of the 1990s must be able to meet and accommodate the rapid evolution of modern-day technology. One such area is laboratory automation. Total automation may be seen as the coupling of computerized sample tracking, electronic documentation and data reduction with automated sample handling, preparation and analysis, resulting in a complete analytical procedure with minimal human involvement. Requirements may vary from one laboratory or facility to another, so the automation has to be flexible enough to cover a wide range of applications, and yet fit into specific niches depending on individual needs.*

*Total automation must be planned for, well in advance, if the endeavour is to be a success. Space, laboratory layout, proper equipment, and the availability and access to necessary utilities must be taken into account. Adequate training and experience of the personnel working with the technology must also be ensured. In addition, responsibilities of installation, programming maintenance and operation have to be addressed. Proper time management and the efficient implementation and use of total automation are also crucial to successful operations.*

*This paper provides insights into laboratory organization and requirements, as well as discussing the management issues that must be faced when automating laboratory procedures.*

### Introduction

The constantly changing world of the analytical laboratory imposes high demands on the area of automation to meet the challenges of its technological advances. There are always new, advanced and improved instrumentation, equipment and labware being discovered and becoming available for use.

Being able to totally automate any process or procedure is the goal that is shared by many in various industries employing analytical technology or performing analytical methodology. However, the individual needs, concerns and means may vary from one laboratory to another. Therefore, the automation must be sufficiently flexible to encompass many applications, and should nevertheless be able to fill specific niches depending on individual requirements.

Just as robotics may have been the buzzword of the mid to late 1980s, total automation will become the new phrase of the 1990s, with everyone automating some, if not all, of their operations.

### Reasons for automation

The reasons for automating the laboratory, especially sample preparation procedures, which according to many are still the major bottleneck in the analytical process, are many and may also vary from laboratory to laboratory or industry to industry. Some reasons which are often thought of initially as important are such things as being able to spend large amounts of money in order to obtain new, expensive and fancy 'toys with lots of bells and whistles' to play with, being the topic of conversation among your peers/colleagues as 'the ones with the robots', and even the prestige of being on every tour making its way through the facility. However, it is the ultimate standardization of procedures facility- and/or industry-wide, increased productivity within the laboratory of working group; improved quality of the resulting generated data through homogeneous sample treatment and better audit trail; simplified method transfer (both internally and externally); multiple operation and application use without retraining and validating each technician for every method or procedure, and improved or better ensured safety of your staff, especially in an area handling large numbers of biological samples; that are all valid and justifiable reasons to automate.

### Definition of total automation

The modern analytical laboratory will be a blend of three approaches to automation. The first type is the one with a fixed geometry and preset programming capabilities, such as autopipetters, autosamplers, etc. The second type are those with a fixed geometry, but with some limited user-programming interactions possible, such as automated workstations. The last type of automation is the user-selected geometry and user-programmed system which is flexible, like the laboratory robotics systems on the market at the present time.

Total automation may be seen as the combination of computerized sample tracking (from receipt through to analysis), electronic documentation and data reduction (through to presentation of the results in a final report format), with automated sample handling, sample preparation (extraction, concentration etc.), and sample analysis (through to the analytical instrumentation), resulting in a complete analytical process with minimal, if any, human involvement. The ideal situation would be to 'phone in through a modem and initiate the operation through voice command.

These integrated systems would increase productivity and improve data integrity, linking the data generator (being workstations and robotics), with the data analyser

© Zymark Corporation 1992

(the instrumentation), and finally data storage (computers, hard drives, floppy disks, tapes etc.). However, the scientist is still the interpreter and an integral and important part of a successful and complete operation. The use of laboratory automation frees the scientists to more extensively analyse and review the resulting data, and interpret the results and their impact on the overall development process. However, one must be careful to avoid getting bogged down in too much data, thereby just transferring the process bottleneck further down the system path, since testing/analysis becomes much easier and relatively quicker when a procedure is automated.

### Planning for automation

Total automation must be planned for well in advance, if it is to be a total success. Considerations must be made for such things as available space (square footage), the laboratory layout (doors, windows, desks, benches—stationary or movable, etc.), appropriate equipment to be able to perform the required application, and the availability and capacity of and access to any necessary services or utilities. Adequate training and experience of the personnel working with the technology must also be ensured, and time must be allocated to allow for this process—not only formal training courses, but also real experience with the equipment/systems. In addition, the responsibilities of system installation, programming, maintenance and final routine operation have to be addressed, with everyone having a clear picture of what is to be expected of them as part of the team to get this project off the ground, and accountability must be assigned and followed through on. Proper time management and the efficient implementation, validation and use of the automated technology are also crucial to successful operations. Finally, certain management issues must be faced and properly dealt with when automating laboratory procedures, so that the automation manager must 'wear many hats' if he/she is to do the job properly. In the 1990s, it may be more important and beneficial in the long run for the manager to be a good businessman, salesman, and public spokesman, rather than just a scientist or engineer.

### Automation considerations

As far as physical dimensions of the automated laboratory are concerned, considerations should be given, in the planning stages if possible, to the available space or square footage, the required layout to properly and efficiently use the automated technology, the proper and necessary equipment to achieve the end goal and its availability from outside vendors, their accessibility and co-operation, or possible necessary in-house resource expenditures in order to make something not available on the market for purchase, and the availability, capacity restraints and ready access to utilities and/or services (such as electrical outlets, gas hook-ups, vacuum etc.). Most laboratories were originally designed and laid out for people (technicians) doing manual sample preparation and analysis. Laboratories used specifically for automated equipment might be better laid out with services being accessed from either above (through the ceiling) or below (through the floor), with the robotics systems, their necessary periphery and analytical instrumentation on mobile carts, tables or islands, giving flexibility for system access, interfacing and possible reconfiguration and redesign, with minimal interruption to the work schedule of the laboratory.

### Successful operation

Another necessary ingredient for the total automation operation to be a success is as a 'manager' of automation: the problem to be solved or goal to be attained with its known limits must be defined; the requirements necessary to achieve this goal must be known and all possible solutions must be investigated; and proven chemistries must be used. The technology should not be made to conform to manual means, but, instead, the technology should advance as a result of its own unique capabilities and ways of doing things. The manager must be proactive, not reactive.

### Personnel issues

An area that has been mentioned to various degrees, and one that is of major importance, is the issue of personnel. A special kind of person is needed to be an automation specialist, with a working knowledge of not only science, but also computers, engineering, mechanics/machinery, electricity, plumbing, and a love for tinkering. The availability of qualified personnel is limited, and will continue to decrease if one pays attention to recent statistics and concerns in the academic world over future supply of basic science majors. Therefore one must ensure the adequate training of the personnel involved with these new technologies through structured education if they are to be successful, and more importantly, one must allow for the 'learning curve' or knowledge gained through experience which only comes with practising the skills learned through hands-on work over a period of time. The people assigned to automation of the laboratory should be dedicated to the task and not job-sharing. A problem in our society and work environments is that we tend to focus on the short term, rather than looking down the road, never leaving enough time to properly achieve the end goal. People should be held accountable for their work, and rewarded for a job well done. As a manager, one is faced with the task of creating a more invigorating work environment, where people are not threatened by the incorporation of automation in the laboratory, but are challenged to advance their careers along new avenues and paths now open to them.

### Validation

Validation issues must also be considered. The implementation and validation of the individual laboratory operations, validation of the entire robotics system or automated workstation, validation of the method or analytical procedure being automated, and validation of the final serialized process (multiple sample preparation or analysis) must be performed. Routine quality control checks and periodic revalidation according to standard operating procedures (SOPs) for regulatory requirements and traceability are important and need to be carried out.

### Management issues

The management issues to be faced are summed up in the following quotation:

> ...we are faced with ever more demanding tasks, to be completed by fewer people, with less funding and shorter lead times, making proper time and resource management of paramount importance in getting the job done in an efficient yet high-quality manner. *The Exceptional Manager.*

The automations manager must address the proper selection of the equipment and vendor, interact with the vendors and establish a good working relationship where everyone benefits, and sometimes be willing to allocate resources of time and money to embark on R&D efforts in-house if the technology needed is not readily available or applicable. The equipment should be goal driven—*the equipment should not drive the goals.* The cost effectiveness and productivity gains of a technology must be evaluated.

A modern-day automations manager can consider the use of 'flexitime' schedules to accommodate the use of automation in the laboratory of the future, allowing personnel to work hours that would make the most efficient use of the robotics systems and automated workstations, rather than force machines and the people who run them into a nine-to-five schedule.

Finally, a good automations manager should train multiple end-users or automation specialists and allow time for them to become fully proficient in the system use, thus establishing an automation team with good synergy, adequate overlap of expertise and a continuity so that the operation does not falter and lose momentum, or worse, come to a halt if someone is transferred, reassigned or leaves.

### Management commitment

It is evident that management has already made a commitment to automation in the research and development environments, and it has been proven that automation, in the almost 10 years of history and experience in the laboratory, is a valuable part of that environment. Laboratory automation is an integral part of today's higher-level business strategy, creating a 'strategic business advantage' (*F. H. Zenie,* Zymark Corporation).

If we could come up with a mission statement such as 'quality science through a commitment to teamwork and excellence', we could correlate this to the research environment, the analytical laboratory, automation and management. There is a commitment to automation that has been demonstrated by many upper-level management committees in various industries, and if we (people and machines) work as a team, we can achieve excellence and produce quality science.

